# Multiplex genome editing of microorganisms using CRISPR-Cas

**DOI:** 10.1093/femsle/fnz086

**Published:** 2019-05-14

**Authors:** Belén Adiego-Pérez, Paola Randazzo, Jean Marc Daran, René Verwaal, Johannes A Roubos, Pascale Daran-Lapujade, John van der Oost

**Affiliations:** 1 Laboratory of Microbiology, Wageningen University and Research, Stippeneng 4, 6708 WE Wageningen, The Netherlands; 2 Department of Biotechnology, Delft University of Technology, Van der Maasweg 9, 2629 HZ Delft, The Netherlands; 3 DSM Biotechnology Center, Alexander Fleminglaan 1, 2613 AX Delft, The Netherlands

**Keywords:** CRISPR-Cas, genome editing, multiplex, cell factories, Cas9, Cas12a

## Abstract

Microbial production of chemical compounds often requires highly engineered microbial cell factories. During the last years, CRISPR-Cas nucleases have been repurposed as powerful tools for genome editing. Here, we briefly review the most frequently used CRISPR-Cas tools and describe some of their applications. We describe the progress made with respect to CRISPR-based multiplex genome editing of industrial bacteria and eukaryotic microorganisms. We also review the state of the art in terms of gene expression regulation using CRISPRi and CRISPRa. Finally, we summarize the pillars for efficient multiplexed genome editing and present our view on future developments and applications of CRISPR-Cas tools for multiplex genome editing.

## Teaser

Current status of multiplex genome editing in bacteria and eukaryotic microorganisms using CRISPR-Cas tools.

## INTRODUCTION

Industrial microbiology plays a key role in the transition towards a more sustainable industry to produce food and feed ingredients, bio-based materials, biofuels and direct synthesis of cosmetic and pharmaceutical compounds (Lee *et al*.^2019^). Oil-based production processes are gradually being substituted by bio-based processes, in which genetically engineered microorganisms are generally crucial to achieve cost-effective productivities and yields (Hong and Nielsen 2012; Dai and Nielsen 2015). The implementation of CRISPR-Cas tools has revolutionized genome editing and mitigated the investment in the metabolic engineering programs required to generate highly engineered microbial cell factories (Donohoue, Barrangou and May 2018; Choi *et al*. 2019).

CRISPR-Cas systems (Clustered Regularly Interspaced Short Palindromic Repeats and CRISPR-associated proteins) are bacterial and archaeal adaptive immune defence systems, which can be repurposed as versatile genetic editing or regulation tools in a broad range of organisms. The effector endonucleases of these systems are guided by short RNA molecules encoded by CRISPR arrays. Native CRISPR arrays consist of a succession of spacers originating from invader organisms separated by direct repeats (Mojica *et al*. 2005; Barrangou *et al*. 2007). Transcription of the CRISPR array results in a long precursor-crRNA transcript (pre-crRNA), that is subsequently being processed to short functional CRISPR RNA (crRNA) guides (Brouns *et al*. 2008). To date, six different types of CRISPR-Cas systems (I–VI) have been described that are divided into two major classes (Class 1 and Class 2) (Makarova and Koonin 2015; Makarova *et al*. 2015; Shmakov *et al*. 2015, 2017; Koonin, Makarova and Zhang 2017). This review focuses on the application of DNA-targeting class 2 CRISPR systems (included in types II and V), that all consist of large multi-domain effector proteins able to use crRNA guides to target complementary DNA. Recent reviews have covered applications of CRISPR-Cas editing in bacteria (Choi and Lee 2016), in *Streptomyces* (Alberti and Corre 2019), in filamentous fungi (Shi *et al*. 2017), in yeast (Stovicek, Holkenbrink and Borodina 2017; Raschmanová *et al*. 2018), in microalgae and cyanobacteria (Naduthodi, Barbosa and Van der Oost 2018), and in general industrial microorganisms (Ferreira, David and Nielsen 2018).

After an introduction of single target genome editing tools, we focus on the spectacular development of multiplexed genome editing by Cas9 (type II) and Cas12a (type V) in industrial microorganisms. Both bacterial and eukaryotic examples are described, although more attention is given to yeast and filamentous fungi, since the diversity of strategies using Cas endonucleases for genome editing applications is more extensive in this group of organisms.

### Single target genome editing and regulation

**Figure 1.**
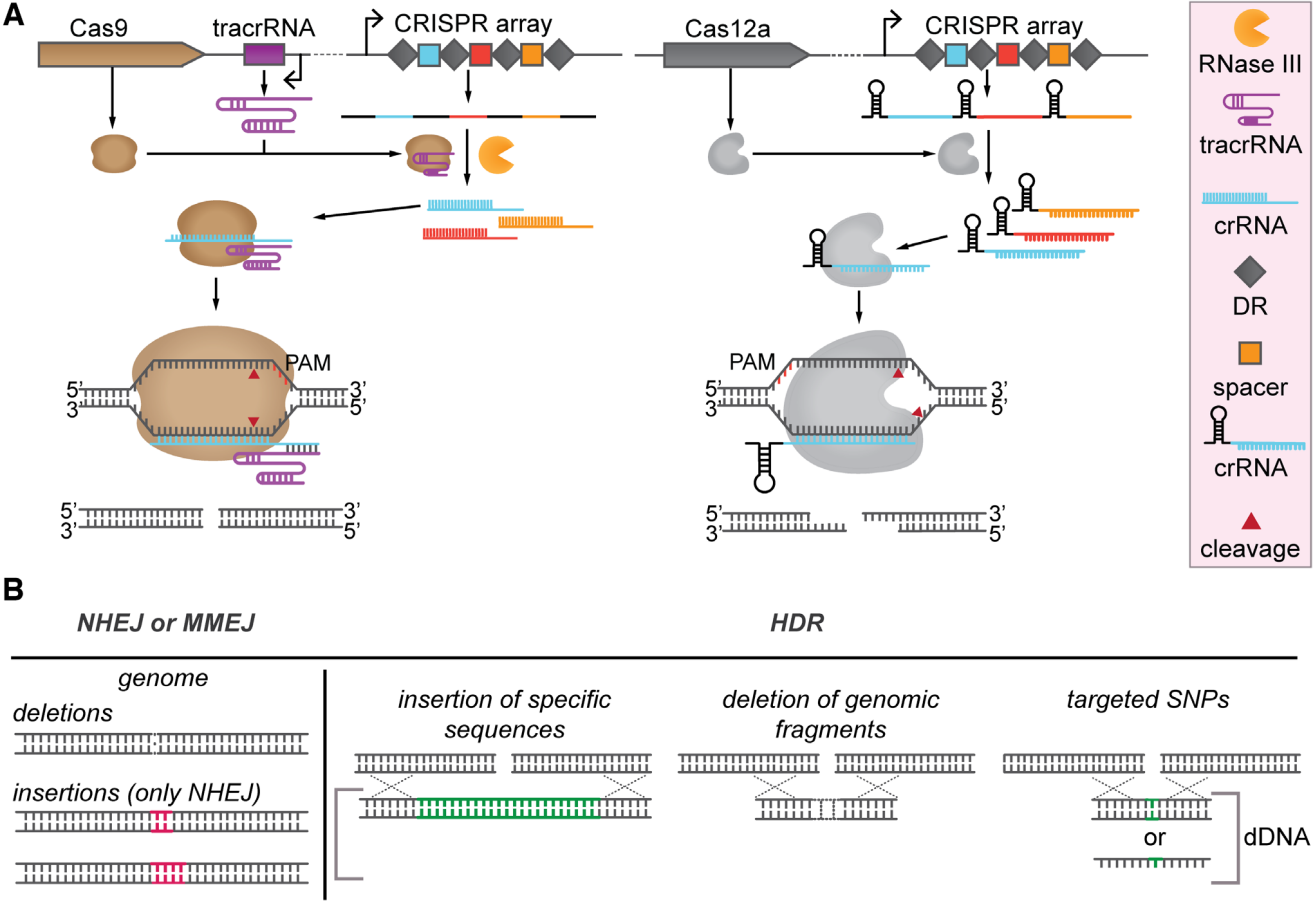
**(A)** Cas9 and Cas12a expression and cleavage schemes. Left panel: Cas9 requires tracrRNA transcription and RNase III expression for CRISPR array transcript processing. Cas9 forms a complex with crRNA and tracrRNA and cleaves target DNA generating blunt ends. Right panel: Cas12a processes its own CRISPR array transcript to obtain individual crRNAs without the requirement of any tracrRNA or RNAse III co-expression. Cas12a stays in complex with crRNA and cleaves target DNA generating staggered ends. **(B)** Double strand break (DSB) repair mechanisms. DSBs can be repaired via non-homologous end joining (NHEJ), alternative non-homologous end joining repair pathways such as microhomology-mediated end joining (MMEJ), or via homologous direct recombination. NHEJ and MMEJ repair pathways can lead to the incorporation of deletions or insertions (only in case of NHEJ) in the targeted region. HDR is combined with the supplementation of donor DNA (dDNA), which can be double stranded or single stranded. dDNA can be used for insertion of long DNA sequences, deletion of genomic fragments, or introduction of single point mutations (SNPs).

Since its establishment as a genome editing tool (Gasiunas *et al*. 2012; Jinek *et al*. 2012), Cas9 from *Streptococcus pyogenes* (*Sp*Cas9) has become the most widely used RNA-guided endonuclease for genome editing and transcription regulation purposes (Table 1). Expression of this type II Cas nuclease together with a guide RNA (gRNA) is sufficient for generating targeted blunt double-stranded breaks (DSBs). The gRNA bound by *Sp*Cas9 consists of two small RNA molecules: a CRISPR RNA (crRNA) and a trans-activating CRISPR RNA (tracrRNA). To simplify gRNA expression, a synthetic chimeric construct named single guide RNA (sgRNA) can be synthesized by fusing the tracrRNA and the crRNA (Jinek *et al*. 2012). Targeting of complementary DNA sequences (protospacers) by the Cas9:gRNA complex requires a protospacer adjacent motif (PAM), in case of Cas9 positioned downstream of the target sequence (Deveau *et al*. 2008). Correct PAM identification and base-pairing will trigger cleavage of the non-target and target DNA strands by the RuvC and HNH nuclease domains, respectively (Gasiunas *et al*. 2012; Jinek *et al*. 2012) (Fig. 1).

**Table 1.** Characteristics of the most commonly used Cas orthologues for genome editing.

	Cas9	Cas12a		
Ortholog	SpCas9	*Fn*Cas12a	*As*Cas12a	*Lb*Cas12a
Subtype	II-A	V-A		
Organism of origin	*Streptococcus pyogenes*	*Francisella novicida*	*Acidaminococcus sp*.	*Lachnospiraceae bacterium*
Nuclease domain	HNH, RuvC	RuvC		
tracrRNA	Yes	No		
PAM (5′–3′)	NGG	TTTV	TTTV	TTTV
Size (amino acids)	1368	1302	1307	1228
RNA processing	No/RNaseIII	Yes/WED III	Yes/WED III	Yes/WED III
Minimum guide length (mature)	∼100 nt	∼44 nt		
Reference(s)	(Deltcheva *et al*. 2011)	(Zetsche, Heidenreich and Mohanraju *et al*. 2017)		

The more recently characterized endonuclease Cas12a (formerly called Cpf1) (type V) can cleave dsDNA directed by a crRNA, hence without the requirement of a tracrRNA (Zetsche *et al*. 2015) (Table 1). Cas12a does not possess an HNH domain, and its RuvC domain has been demonstrated to cleave both the non-target and the target DNA strands (Swarts, Van der Oost and Jinek 2017; Swarts and Jinek 2018). Moreover, Cas12a is able to process its crRNA guide autonomously (Fonfara *et al*. 2016; Swarts, van der Oost and Jinek 2017; Zetsche *et al*. 2017), while Cas9 relies on the activity of an additional non-Cas, dsRNA (crRNA/tracrRNA) targeting ribonuclease (RNaseIII) (Deltcheva *et al*. 2011). Both Cas9 and Cas12a can use multiple crRNA guides for creating simultaneous DSBs at different target loci in the genome (Fig. 1). Recently, two distinct Cas12 subtypes (Cas12b, CasX/Cas12e) were shown to also edit genomes of bacteria and mammalian cells (Liu *et al*. 2019; Strecker *et al*. 2019).

Genomic DSBs can be repaired by homology-directed repair (HDR), non-homologous end joining repair (NHEJ) or alternative non-homologous end joining systems such as microhomology-mediated end joining (MMEJ) (Chayot *et al*. 2010; Sfeir and Symington 2015; Yao *et al*. 2017). The error-prone NHEJ repair system is often most prevalent in eukaryotes (Pawelczak *et al*. 2018) (Fig. 1), whereas it has been predicted to be encoded by only ∼26% of publicly available prokaryotic genomes (Bowater and Doherty 2006; McGovern *et al*. 2016; Nayak and Metcalf 2017). The less studied alternative MMEJ repair system has been reported to also be present in bacteria and fungi (Sfeir and Symington 2015). This repair system has been proven to be active together with other repair mechanisms in the fungi *Aspergillus niger* and *Yarrowia lipolytica* (Shi *et al*. 2018) or in NHEJ-free bacteria (Chayot *et al*. 2010). HDR can be used in a targeted way: (i) to insert DNA fragments in targeted genomic locations; (ii) to delete small and large DNA fragments; or (iii) to introduce point mutations. HDR requires the introduction of a single or double-stranded DNA repair fragment into the cell, called donor DNA (dDNA), encoding the desired novel property or designed nucleotide change. To avoid targeting after the designed change, the recombinant sequence generally contains one or more silent mutations in the protospacer or PAM recognition sequence or partial deletion thereof. On the other hand, in organisms with highly active NHEJ or MMEJ repair systems, the introduction of non-specific insertions (only in case of NHEJ) and/or deletions (indels) in a certain target sequences can lead to gene disruption (Cong *et al*. 2013).

Inactive or deactivated versions of both Cas9 and Cas12a (named dCas9 and dCas12a) have been designed by substituting one or more of the catalytic amino acids in the nuclease domains (Gasiunas *et al*. 2012; Jinek *et al*. 2012; Zetsche *et al*. 2015; Swarts and Jinek 2018). These variants have been used to regulate gene expression in many organisms since they retain the target-binding ability (Berlec *et al*. 2018). By directing dCas9 or dCas12a to the promoter or coding sequence of a target gene, transcriptional repression (silencing) can be achieved by steric hindrance of the RNA polymerase and/or of transcription factors required for transcription of the target gene. This CRISPR interference (CRISPRi) technique has initially been established in *Escherichia coli*, resulting in significant transcriptional repression when targeting either the promoter or the non-template DNA strand of an open reading frame (Bikard *et al*. 2013; Qi *et al*. 2013). In eukaryotic microorganisms, gene repression is normally achieved by fusing repressor domains such as the mammalian transcriptional repressor domain Mxi1 or the Krüppel-associated box (KRAB domain) to the C-terminus of dCas9 or dCas12a (Jensen *et al*. 2017; Jensen 2018). Recently, native repression domains have been characterized in the yeast *Saccharomyces cerevisiae* with multiple Cas9 orthologs (Lian *et al*. 2017). This practice is more common in eukaryotic organisms since the use of only a dCas9:gRNA-complex seems not to be sufficient to significantly block transcription (Gilbert *et al*. 2013). Moreover, fusions of these deactivated variants to transcription activation domains are used to achieve gene activation (CRISPRa). In eukaryotes, VP16, VP64, Gal4^AD^ or the synthetic VPR activator domains have been used successfully (Chavez *et al*. 2015; Jensen *et al*. 2017; Schwartz *et al*. 2018), while the omega (ω) subunit of the RNA polymerase has been used in bacteria (Bikard *et al*. 2013; La Russa and Qi 2015).

### Multiplex genome editing

Editing of multiple loci is often required to introduce multiple heterologous genes and to fine-tune metabolic networks of microbial cell factories. In the pre-CRISPR era, iterative rounds of genome editing making use of selection markers were necessary to build strains expressing multiple-gene expression pathways. Establishment of marker-free CRISPR-Cas tools brought powerful nuclease-mediated multiplex genome engineering capabilities, considerably saving time and resources in strain construction programs. The multiplexing capabilities of CRISPR-Cas systems as genome editing tools have been widely exploited with Cas9 and more recently with Cas12a. One of the first microbial applications demonstrating the multiplexing capabilities of Cas9 was performed with the native Cas9 system of *Streptococcus pneumoniae* and two spacers expressed from a synthetic array integrated into the genome (Jiang *et al*. 2013). After this proof of principle, multiple studies explored the multiplexing capabilities of the endonuclease Cas9 in mammalian cells (Cong *et al*. 2013; Mali *et al*. 2013), as well as in industrially relevant prokaryotic and eukaryotic microorganisms (Table 2).

**Table 2.** Multiplexed genome editing events in industrial microorganisms using CRISPR-Cas systems.

*Specie* [strain(poidy)]	Cas nuclease tool (expression), Plasmid (replication origin)/genome integrated	Strategy for multiplexed gRNA expression/delivery (expression), plasmid (replication origin)/genome integrated	Type of donor DNA: HFs;amount/concentration	Type of modification: Number of target, editing efficiency	Reference
**PROKARYOTES**					
*Escherichia coli*[MG1655]	*Sp*Cas9 (inducible),Plasmid expression (repA101 ori)	Several sgRNA expression cassettes (constitutive),Plasmid expression	Circular dsDNA (pMB1 ori):∼300 bp	Knockouts: 2, 100%; 3, 88.3%; 4, >30%	(Feng *et al*. 2018)
*Escherichia coli*[MG1655]	*Sp*Cas9 (constitutive),Plasmid expression (repA101 (Ts) ori)	Several sgRNA expression cassettes (constitutive),Plasmid expression (pMB1 ori)	Circular dsDNA (pMB1 ori): 250–550 bp	Knockouts: 2, 97% ± 4%; 3, 47% ± 8%	(Jiang *et al*. 2015)
*Escherichia coli*[MG1655]	*Sp*Cas9 (inducible),Plasmid expression (ColE1 ori)	Several sgRNA expression cassettes (inducible),Plasmid expression (pMB1 ori)	Linear ssDNA: 70 bp; 5 pmol	Short insertions: 2, ∼70%	(Ronda *et al*. 2016)
*Escherichia coli*[MG1655]	*Sp*Cas9 (constitutive),Plasmid expression (p15A ori)	Several sgRNA expression cassettes (constitutive),Plasmid expression (ColE1 ori)	Linear ssDNA: ∼89 bp; 50 pmol	Point mutations: 2, 83%; 3, 23%	(Li *et al*. 2015)
*Streptococcus pneumoniae*[crR6c]	*Sp*Cas9 (constitutive),Genome integrated	Native-like CRISPR array (constitutive),Genome integrated	Linear dsDNA: not mentioned ;0.7 ng/µl to 2.5 µg/µl	Deletions: 2, 75%	(Jiang *et al*. 2013)
*Streptomyces lividans*	*Sp*Cas9 (constitutive),Plasmid expression (pSG5rep)	Several sgRNA expression cassettes (constitutive),Plasmid expression (pSG5rep)	Circular dsDNA (oriT): 1 kB	Short deletions (20–34 bp): 2, 100% (4/4)	(Cobb, Wang and Zhao 2015)
*Streptomyces coelicolor*[M145]	*Sp*Cas9 (constitutive),Plasmid expression (pSG5rep)	Several sgRNA expression cassettes (constitutive),Plasmid expression (pSG5rep)	Circular dsDNA (pSG5): ∼1 kB	Deletions (768–1053 bp): 2, 29–54%	(Huang *et al*. 2015)
*Escherichia coli*[MG1655]	*Fn*Cas12a (inducible),Plasmid expression (repA 101)	Native-like CRISPR array (constitutive),Plasmid expression (pSC101 ori)	Circular dsDNA (oriE): 500 bp	Gene insertions: 3, ∼20%	(Ao *et al*. 2018)
*Streptomyces coelicolor*[M145]	*Fn*Cas12a (constitutive),Plasmid expression (pSG5rep)	Native-like CRISPR array (constitutive),Plasmid expression (pSG5)	Circular dsDNA (pSG5): ∼1 kB	Knockouts: 2, 75%	(Li *et al*. 2018)
**EUKARYOTES**					
*Saccharomyces cerevisiae*[CEN.PK113–7D (n), CEN.PK2–1c (n), CEN.PK122 (2n)]	*Sp*Cas9 (constitutive),Genome integrated	Several sgRNA expression cassettes (RNA pol III promoter, constitutive),Plasmid expression (multicopy)	Linear dsDNA: 60 bp; 12 pmols	Knockouts: 2, 100%; 6, 65%	(Mans *et al*. 2015)
*Saccharomyces cerevisiae*[CEN.PK2–1c (n)]	*Sp*Cas9 (constitutive),Genome integrated	Several sgRNA expression cassettes with homology flanks to a linearized plasmid backbonePlasmid expression (multicopy)	Linear dsDNA: 500 bp; 0.6–1.54 pmols	Knockouts: 3, 64%	(Horwitz *et al*. 2015)
*Saccharomyces cerevisiae*[CEN.PK113–5D (n)]	*Fn*Cpf1 (constitutive),Genome integrated	Native-like CRISPR array (RNA pol III promoter, constitutive)Plasmid expression (multicopy)	Linear dsDNA: 60 bp; 12 pmols	Knockouts: 2, 100%; 4, 85%	(Swiat *et al*. 2017)
*Saccharomyces cerevisiae*[CEN.PK113–7D (n), Ethanol Red (2n)]	*Sp*Cas9 (constitutive),Plasmid expression (multicopy)	Several sgRNA expression cassettes (RNA pol III promoter, constitutive),Plasmid expression (multicopy)	Linear ssDNA: 40 bp; 300pmols	Knockouts: 2, 91–98%	(Generoso *et al*. 2016)
*Saccharomyces cerevisiae*[BY4741 (n)]	*Fn*Cpf1 (constitutive),Plasmid expression (centromeric)	Native-like CRISPR array (RNA pol III promoter, constitutive),Plasmid expression (multicopy)	Linear dsDNA: 50 bp	Multi-gene integrations: 2, 52%; 3, 43%	(Li, Wang and Wei 2018)
*Saccharomyces cerevisiae*[204 508; ATCC (mated) (2n)]	*Sp*Cas9 (constitutive), Plasmid expression (multicopy)	Synthetic array of ribozyme-flanked sgRNA (RNA pol III promoter, constitutive),Plasmid expression (multicopy)	Linear dsDNA: 50 bp; 44.94 pmol	Deletions: 2, 43%; 3, 19%	(Ryan *et al*. 2014)
*Saccharomyces cerevisiae*[CEN.PK113–7D (n)]	*Lb*Cpf1, *As*Cpf1 or *Fn*Cpf1 (constitutive), Plasmid expression (centromeric)	Native-like crRNA-array with homology flanks to a linearized plasmid backbone (RNA pol III promoter, constitutive),Plasmid expression (multicopy)	Linear dsDNA: 50 bp; 90–120 fmols	Multi-gene integrations: 3, 91%	(Verwaal *et al*. 2018)
*Saccharomyces cerevisiae*[BY4741 (n), CEN.PK2–1c (n)]	i*Sp*Cas9 (constitutive),Plasmid expression (multicopy)	Native-like crRNA-array (RNA pol III promoter, constitutive), separate expression of tracrRNA,Plasmid expression (multicopy)	Circular dsDNA, at 5’ of each spacer sequence (multicopy): 50 bp; 142 fmols	Knockouts: 3, 27–87%	(Bao *et al*. 2015)
*Saccharomyces cerevisiae*[CEN.PK113–7D (n)]	*Sp*Cas9 (constitutive),Genome integrated	Synthetic crRNA-array (RNA pol III promoter), separate expression of *Pa*Csy4, Plasmid expression (multicopy)	Linear dsDNA: 60 bp; 12 pmols	Knockouts: 2, 100%; 4, 96%	(Ferreira, *et al*. 2018)
*Saccharomyces cerevisiae*[BY4741 (n)]	*Sp*Cas9 (constitutive),Plasmid expression (centromeric)	Several HDV ribozyme-sgRNA expression cassettes (RNA pol III promoter, constitutive),Plasmid expression (centromeric)	Linear dsDNA, barcoded: 60 bp; 55 pmols	Knockouts: 2, 65–87.5%; 3, 57.5–75%; 4, 27.5–15%	(Lee *et al*. 2015)
*Saccharomyces cerevisiae*[CEN.PK2–1C (n)]	*Sp*Cas9 (constitutive),Plasmid expression (centromeric)	Several sgRNA expression cassettes (RNA pol III promoter, constitutive),Plasmid expression (multicopy)	Linear dsDNA: 500 bp; 700 fmols	Multi-gene integrations: 3, 84%	(Ronda *et al*. 2015)
*Saccharomyces cerevisiae*[CEN.PK111–27B (n)]	*Sp*Cas9 (constitutive),Plasmid expression (centromeric)	Several sgRNA expression cassettes (RNA pol III promoter, constitutive),Plasmid expression (multicopy)	Linear dsDNA: 50 bp; 4 pmols	Multi-gene integrations: 2, 58%; 3, 30.6%	(Jakočiūnas *et al*. 2015)
*Saccharomyces cerevisiae*[CEN.PK2–1C]	*Sp*Cas9 (constitutive),Genome integrated	Several sgRNA expression cassettes (RNA pol III, constitutive), some target more than one site,Plasmid expression (multicopy)	Linear dsDNA: 60 bp; 26.96 pmol	Deletions: 9, 50% (only 2 transformants on plate)	(Wijsman *et al*. 2019)
*Saccharomyces cerevisiae*[CEN.PK 113–5D]	*Sp*Cas9 (constitutive),Multicopy plasmid	Synthetic crRNA-array (with one RNA pol III promoter for the expression of four gRNAs), gRNAs between tRNA^gly^ sequences, Plasmid expression (multicopy)	Linear dsDNA: 50 bp; 266.9 pmol	Deletions (8 bp): 8, 86.7%	(Zhang *et al*. 2019)
*Ogataea parapolymorpha*[CBS 11 895 (n)]	*Sp*Cas9 (constitutive),Plasmid expression (centromeric)	Synthetic array of ribozyme-flanked sgRNA (RNA pol II promoter, constitutive),Plasmid expression (centromeric)	Linear dsDNA: 480 bp, 1.6 pmols	Knockouts: 2, 2–5%	(Juergens *et al*. 2018)
*Ogataea polymorpha*[CGMCC7.89 (n)]	i*Sp*Cas9 (constitutive),Genome integrated	Several sgRNA expression cassettes expressed (RNA pol III promoter, constitutive),Genome integrated	Linear dsDNA: 1 kb, 1.7 pmols	Multi-gene integrations: 3, 30.56 ± 2.40%	(Wang *et al*. 2018a)
*Yarrowia lipolytica*[ATCC 201 249 (n), ATCC MYA-2613 (n)]	*Sp*Cas9 (constitutive),Plasmid expression (centromeric)	Synthetic array of ribozyme-flanked sgRNAs (RNA pol II promoter, constitutive),Plasmid expression (centromeric)	On multicopy plasmid: ∼450bp	Knockouts: 2, 36.7 ± 8.5%; 3, 19.3 ± 9.2%	(Gao *et al*. 2016)
*Penicillium chrysogenum*[DS68530]	*Sp*Cas9 (transient), Delivered as a RNP	*In vitro* synthetized sgRNA in RNP, protoplast-mediated transformation, Transient expression	Linear dsDNA: ≥ 1 kb, 1–11 µg	Cassette integration: 2, 50%	(Pohl *et al*. 2016)
*Aspergillus nidulans*[IBT27263 (n)]	*Sp*Cas9 (constitutive), Plasmid expression (centromeric)	Synthetic array of tRNA-flanked sgRNAs (RNA pol III promoter, constitutive),Plasmid expression (centromeric)	Linear ssDNA: 45 bp, 1 µmol	Multi-purpose: 3, 90%	(Nodvig *et al*. 2018)
*Trichoderma reesei*[ATCC 13 631, (n); ATCC 56 765 (n)]	*Sp*Cas9 (inducible), Genome integrated	*In vitro* synthetized sgRNA delivery by protoplasts transformation, Transient expression	Linear dsDNA: 200 bp, 296 pmols	Knockouts: 2, 16–45%; 3, 4.2%	(Liu *et al*. 2015)
*Scheffersomyces stipitis*[UC7, (n)]	*Sp*Cas9 (constitutive), Plasmid expression (centromeric)	Several sgRNA expression cassettes (RNA pol III promoter, constitutive),Plasmid expression (centromeric)	Linear dsDNA: 500 bp	Knockouts: 2, 40%	(Cao *et al*. 2018)
*Kluyveromyces lactis*[ATCC8585 (n)]	*Sp*Cas9 (constitutive),Genome integrated	Several sgRNA expression cassettes with homology flanks to a linearized plasmid backbone,Plasmid expression (multicopy)	Linear dsDNA: 1 kb, 0.6–1.54 pmols	Multi-gene integration: 3, 2.1%	(Horwitz *et al*. 2015)
*Myceliophthora thermophila*[ATCC 42 464, (n)]	*Sp*Cas9 (constitutive),Plasmid expression (centromeric)	Several sgRNA expression cassettes (RNA pol III promoter, constitutive),Transient expression	Linear dsDNA: 600 bp; ∼12pmols	Knockouts: 2, 61–70%; 3, 30%; 4, 22%	(Liu *et al*. 2017)
*Saccharomyces pastorianus*[CBS1483, (n#)]	*Sp*Cas9 (constitutive),Plasmid expression (centromeric)	Synthetic array of ribozyme-flanked sgRNA (RNA pol II promoter, constitutive),Plasmid expression (centromeric)	Linear dsDNA: 60 bp; 12pmols	Knockouts: 2, 100%	(Gorter de Vries *et al*. 2017)
*Komagataella phaffi*[CBS7435 (n)]	*Sp*Cas9 (constitutive),Plasmid expression (centromeric)	Several sgRNA (ribozyme-flanked) expression cassettes (RNA pol II promoter, constitutive),Plasmid expression (centromeric)	Linear dsDNA: 1 kb; ∼400–770fmols	Knockouts: 2, 69 ± 13%	(Weninger *et al*. 2016)
*Phaeodactylum tricornutum*(2n)	*Sp*Cas9 (transient),Delivered as a RNP	*In vitro* synthetized sgRNA in RNP, biolystic delivery, Transient expression	–	Knockouts: 2, 65–100%; 3, 15.4%	(Serif *et al*. 2018)
*Magnaporthe oryzae*(2n)	*Sp*Cas9 (transient),Delivered as a RNP	*In vitro* synthetized sgRNA in RNP, protoplast-mediated transformation, Transient expression	Linear dsDNA: ∼40 bp	Knockouts by SNP: 2, 3.4–12.3%	(Foster *et al*. 2018)
*Fusarium fujikuroi*[NJtech 02, CCTCC M2015614]	*Sp*Cas9 (constitutive),Plasmid expression (centromeric)	Several sgRNA expression cassettes (RNA pol III promoter, constitutive),Plasmid expression (centromeric)	–	Knockouts by disruption: 2, 20.8%; 3, 4.2%	(Shi *et al*. 2019)

#Anaeuploid.

The crRNA processing activity of the recently characterized Cas12a increases the simplicity of multiplexing. By expressing a single CRISPR array containing multiple spacers under the transcriptional regulation of a single promoter and terminator, multiple loci can be targeted simultaneously (Zetsche *et al*. 2017). Therefore, there is no need of supplying multiple targeting expression constructs. Recent studies have demonstrated the multiplex editing potential of the Cas12a endonuclease in a wide range of microorganisms (Table 2).

Aneuploidy and polyploidy are common conditions among eukaryotic industrial microorganisms. The requirement for simultaneous targeting of multiple alleles in non-haploid strain results in a decrease of the CRISPR editing efficiencies (Mertens *et al*. 2019). The term ‘*cis*-multiplexing’ is used when targeting a single genomic locus found multiple times across the genome of a non-haploid organism. ‘*Trans*-multiplexing’ refers to the simultaneous introduction of modifications in multiple genes that occur in more than one copy in the genome (Ryan *et al*. 2014). Several examples of both *cis*- and *trans-* multiplexing have recently been described (Table 2).

### gRNA expression systems for efficient multiplex genome editing

**Figure 2.**
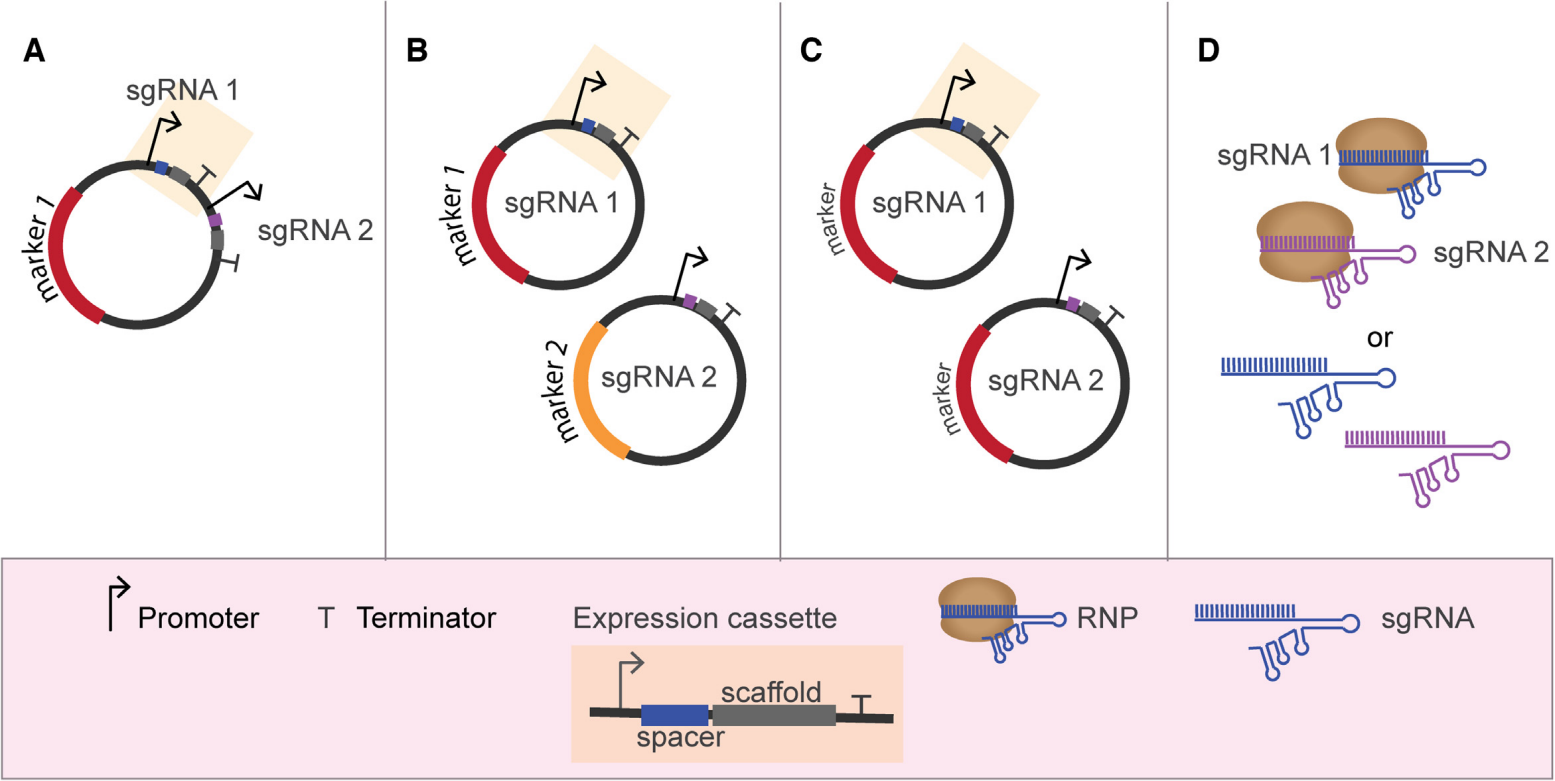
Multiplexing using single gRNA expression cassettes. **(A)** Expression of several sgRNA cassettes from a single expression vector. **(B)** Expression of several sgRNA cassettes from multiple expression vectors (each harboring a different marker). **(C)** Expression of several sgRNA cassettes from multiple expression vectors (all harboring the same marker). **(D)** Transient supplementation with *in vitro* assembled RNPs or *in vitro* transcribed gRNAs.

**Figure 3.**
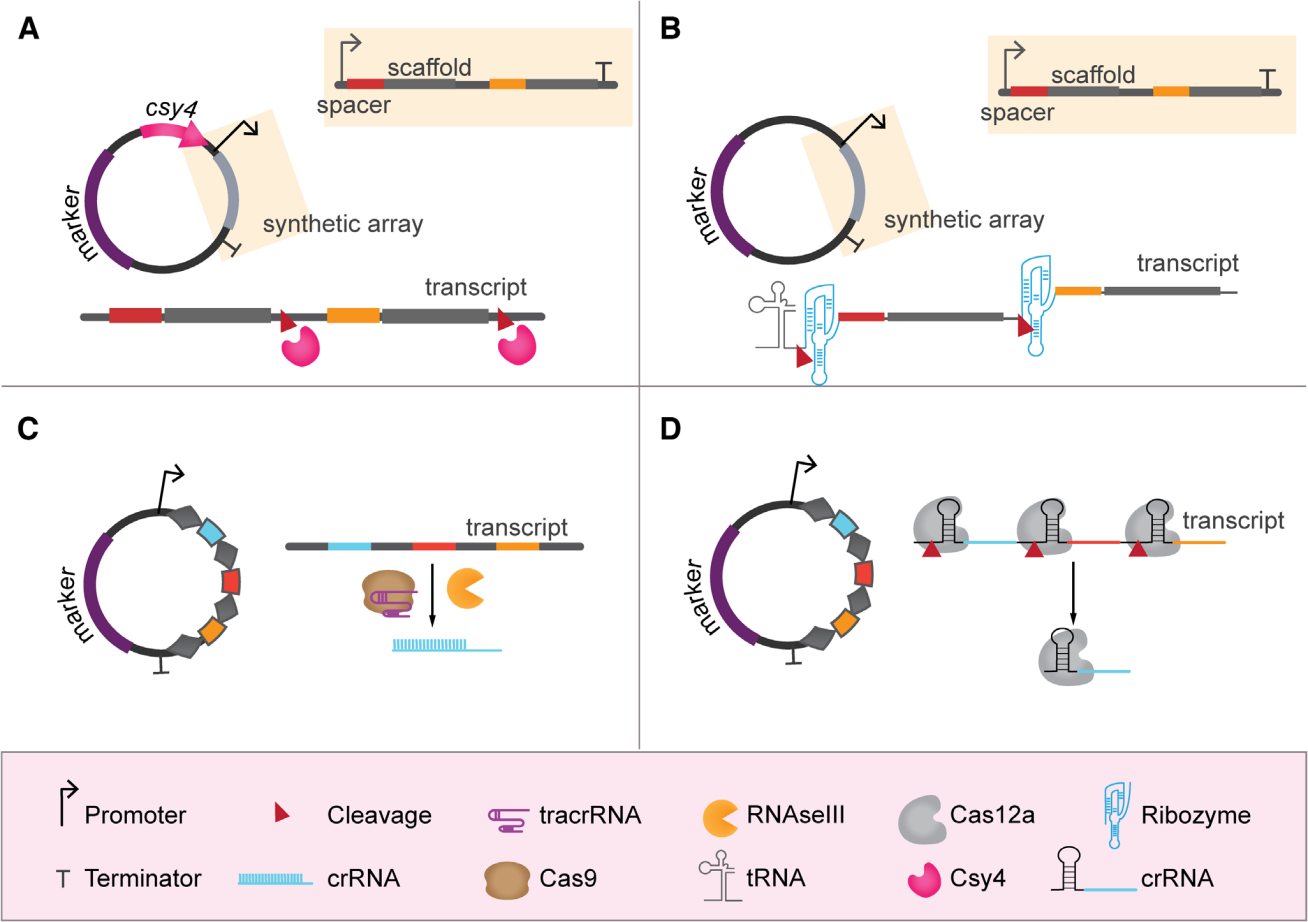
Multiplexing using gRNA polycistronic cassettes. **(A)** Expression of gRNAs from synthetic array dependent on Csy4 processing. In this case, Csy4 has to be co-expressed. **(B)** Expression of gRNAs from synthetic array dependent on endoribonuclease splicing. In most of the reviewed examples, these synthetic arrays are expressed using tRNAs as RNA pol III promoters. **(C)** Expression of gRNAs from native-like CRISPR array dependent on Cas9, tracrRNA and RNAse III processing. **(D)** Expression of gRNAs from native-like CRISPR array dependent on Cas12a processing.

In most reported cases of genome editing of industrial microorganisms, and in all bacterial examples, gRNAs are generally expressed from multicopy plasmids that, after successful editing, can be removed through counter selection (Bao *et al*. 2015; Si *et al*. 2017) or growth on non-selective medium (Mans *et al*. 2015; Wijsman *et al*. 2019). In case of multiplex genome editing, two strategies have been described for the delivery of the different gRNAs: (i) multiple gRNA expression cassettes transcribing a sgRNA molecule from one or more plasmids (Fig. 2); (ii) polycistronic expression of gRNAs, either inspired by native CRISPR systems or by synthetically designed ones (Fig. 3). Individual gRNAs can also be *in vitro* transcribed and supplied directly to filamentous fungi or microalgae (Table 2).

#### Multiplexing using multiple single gRNA expression cassettes

The initial attempts of multiplex genome editing using *Sp*Cas9 relied on combined expression of several individual gRNA expression cassettes. In bacteria, different strength natural or synthetic promoters (constitutive or inducible) are routinely used for gRNA expression (Table 2). In eukaryotes, efficient gRNA expression might be controlled by an RNA polymerase-III-dependent promoter. In a cell that expresses a nuclear-localized *Sp*Cas9, this gRNA can direct the nuclease to its target. In addition, fusions of gRNAs and tRNA auto-splicing sequences (used as promoters) have also been demonstrated to yield multiple functional gRNAs in eukaryotes (Schwartz *et al*. 2016; Song *et al*. 2018). Alternatively, gRNAs flanked with self-cleaving ribozymes on both ends have been expressed from RNA polymerase II-dependent promoters to provide transcripts with modified ends and increase transcript stability in the nucleus (Gao and Zhao 2014; Nødvig *et al*. 2015; Weninger *et al*. 2016; Wong *et al*. 2017). Moreover, expression of a bacterial T7 RNA polymerase in the yeasts *Y. lipolytica* and *Kluyveromyces lactis* has been used for guide expression (Morse *et al*. 2018).

In bacteria, the maximal number of reported simultaneous editing events using *Sp*Cas9 varies from organism to organism (Table 2). Expression of multiple gRNAs from multiple expression cassettes is done from plasmid-borne or genome-integrated constructs. Most reported examples of multiplex genome editing using gRNAs expressed from multiple expression cassettes have been performed in *E. coli* (Jiang *et al*. 2015; Li *et al*. 2015; Ronda *et al*. 2016), with a maximum of four genes targeted simultaneously and an editing efficiency of ∼30% (Feng *et al*. 2018). The authors of the study developed a CRISPR multiplex genome editing technique that uncouples transformation and editing. This separation is achieved by inducing Cas12a expression only after transformation and seems to be key, together with recombineering, for increased editing efficiencies (Reisch and Prather 2015; Feng *et al*. 2018).

In eukaryotes, the combination of several gRNA expression modules in a single amplicon or spread over several co-transformed plasmids resulted in successful editing in several organisms such as *S. cerevisiae* (Horwitz *et al*. 2015; Jakočiūnas *et al*. 2015; Lee *et al*. 2015; Mans *et al*. 2015; Ronda *et al*. 2015; Generoso *et al*. 2016; Deaner, Holzman and Alper 2018), *Komagataella phaffi* (Weninger *et al*. 2016), the xylose-utilizing yeast *Scheffersomyces stipitis* (Cao *et al*. 2018), the enzyme producer *Myceliophthora thermophila* (Liu *et al*. 2017), *K. lactis* (Horwitz *et al*. 2015), *Fusarium fujikuroi* (Shi *et al*. 2019), the methylotrophic yeast *Ogataea polymorpha* (Wang *et al*. 2018a) and the oleaginous yeast *Y. lipolytica* (Gao *et al*. 2016) (Table 2). In many organisms, high editing efficiencies allow for straightforward screening and selection of the desired mutant without introducing selectable markers in their genome. However, the reported efficiencies for targeting two or more sites simultaneously in eukaryotes vary significantly from one organism to another (i.e. from 2 to 100%). Using *Sp*Cas9, nine editing events in *S. cerevisiae* is the highest number of simultaneous modifications reported in microbes to date (Table 2) (Wijsman *et al*. 2019).

In some filamentous fungi, the gRNA molecules can be synthetized *in vitro* and co-transformed with Cas9-encoding plasmids. In microalgae, *in vitro* synthetized gRNAs can be delivered together with *in vitro* produced Cas proteins as ribonucleoprotein complexes (RNPs) as well (Liu *et al*. 2015; Pohl *et al*. 2016; Shi *et al*. 2017; Naduthodi, Barbosa and van der Oost 2018). Simultaneous double editing has been achieved by following this strategy in *Penicillium chrysogenum* (Pohl et al. 2016,2018), in *Phaeodactylum tricornutum* (Serif *et al*. 2018) and in the rice blast fungus *Magnaporthe oryzae* (Foster *et al*. 2018). Although transformation of *in vitro* synthetized gRNA simplifies gRNA cloning work and avoids the requirement of identifying effective RNA polymerase III promoters in eukaryotes, efficient selection of transformants may still require chromosomal integration of selectable markers located in the dDNA molecules (Liu *et al*. 2015; Pohl *et al*. 2016). These markers can be removed from the genome by using counter-selection markers or Cre-recombinase-based approaches.

Finally, although *Sp*Cas9 has been successfully implemented in microalgae, we found few examples for the use thereof for multiplexed genome editing (Behler *et al*. 2018; Naduthodi, Barbosa and van der Oost 2018). Recently, a triple knockout strain of the microalgae *P. tricornutum* was generated in a single step transformation using six different Cas9-based RNP complexes (Serif *et al*. 2018).

#### Multiplexing using gRNA polycistronic cassette

Cas9-dependent multiplex genome editing efforts reported to date are generally based on the combination of individual expression cassettes required for single targeting editing experiments. Hence, they commonly suffer from (i) repeated usage of equal sets of promoters and terminators for gRNA expression, (ii) requirement for multiple selection markers, and more importantly, (iii) labor-intensive expression cassettes and plasmid construction. By expressing multiple gRNAs under the control of a single promoter, polycistronic expression cassettes overcome these hurdles. To date, two polycistronic cassette-based approaches have been shown that enable multiplex genome editing (Fig. 3).

The first approach uses tandem arrays of chimeric sgRNAs that require a dedicated maturation mechanism to release the individual sgRNAs after transcription (Fig. 3A and B). The Csy4 ribonuclease cleaves synthetic precursor sgRNA arrays when interspaced by 28-nt sequences recognized by Csy4 (Qi *et al*. 2012). As such, efficient quadruplex editing by *Sp*Cas9 has been accomplished in *S. cerevisiae* (Ferreira *et al*.^2018^). Alternatively, cleavage of the crRNA array can be performed by self-cleaving RNA sequences. As such, efficient duplex editing was achieved in the non-conventional yeasts *O. parapolymorpha* and *Saccharomyces pastorianus* using sgRNAs flanked with Hammerhead and Hepatitis Delta Virus ribozymes (Gorter de Vries *et al*. 2017; Juergens *et al*. 2018). Triplex editing was achieved in a diploid *S. cerevisiae* strain with a synthetic array of sgRNAs flanked by ribozymes (Ryan *et al*. 2014). In addition, tandem fusion of multiple sgRNAs-tRNAs enabling sgRNAs processing by the native tRNA-maturation system was shown to promote *Sp*Cas9-assisted multiplexed genome editing in filamentous fungi (Nodvig *et al*. 2018).

The second approach for polycistronic gRNA expression resembles native CRISPR arrays (Fig. 3C and D). The expression of Cas9 is required together with the transcription of a CRISPR array with multiple crRNAs and a tracrRNA. With this approach, an array of two guides was expressed and processed in a self-targeting system in *E. coli* for plasmid removal. Processing of the CRISPR array transcript occurred via unknown native endoribonucleases (Ronda *et al*. 2016). Again, resembling native CRISPR systems, a three-spacers array and a tracrRNA were co-expressed in *S. cerevisiae* and up to three genes were deleted with efficiencies ranging from 27–87% (depending on the sequence of the targeting guides) (Bao *et al*. 2015). More recently, several orthologues from the class V endonuclease Cas12a (*As*Cas12a, *Lb*Cas12a and *Fn*Cas12a) (Zetsche *et al*. 2015) were shown to deliver efficient multiplex genome editing of *E. coli* (Ao *et al*. 2018), *Streptomyces coelicolor* (Li *et al*. 2018) and *S. cerevisiae* (Swiat *et al*. 2017; Verwaal *et al*. 2018; Li, Wang and Wei 2018).

### Efficient delivery of donor DNA for repair of CRISPR-mediated DNA breaks

Current multiplexing strategies are based on two different procedures for the simultaneous delivery of dDNA fragments. On one hand, transient delivery of these homology repair templates is accomplished by co-transforming multiple linear dDNAs with a corresponding set of gRNAs. This approach was implemented for efficient introduction of multiple barcoded repairs fragments (short DNA fragments that contain a unique sequence tag) (Ryan and Cate 2014), and editing of heterologous metabolic pathways in both eukaryotic and prokaryotic genomes (Jiang *et al*. 2013; Jakočiūnas *et al*. 2015; Li *et al*. 2015; Mans *et al*. 2015; Ronda *et al*. 2015; Verwaal *et al*. 2018; Wang *et al*. 2018a). While dDNA is often delivered in double-stranded configuration, it should be noted that successful repair using single-stranded DNA (ssDNA) has also been achieved in *S. cerevisiae* (DiCarlo *et al*. 2013; Generoso *et al*. 2016), *Aspergilli* (Nodvig *et al*. 2018), in *E. coli* (Li *et al*. 2015; Ronda *et al*. 2016), and in mammalian cells (Richardson *et al*. 2016; Song and Stieger 2017). Alternatively, a stable source of dDNA sequences is provided when these are cloned in one multi-copy plasmid (Cobb, Wang and Zhao 2015; Huang *et al*. 2015; Jiang *et al*. 2015; Gao *et al*. 2016; Ao *et al*. 2018; Feng *et al*. 2018; Li *et al*. 2018). In a more elegant way, these sequences can be cloned in tandem to the corresponding gRNAs under control of a single promoter. These hybrids are long transcripts that include multiple repair template-crRNA sequences between CRISPR repeats to be further processed into shorter gRNAs. These gRNAs will still include the transcribed sequence of each dDNA template (Bao *et al*. 2015; Garst *et al*. 2017; Roy *et al*. 2018).

Repair efficiencies of the double-stranded DNA break at a targeted locus depend on the size of the homology flanks (HFs) of the dDNA. Commonly used dDNA sequences may vary from short-sized HFs (∼50 bp for *S. cerevisiae* or ∼50–100 bp for *E. coli*), to longer, PCR-based HFs for many bacteria, non-conventional yeasts and fungi (200–1000 bp) (Table 2). Long HFs have been shown to increase the efficiency of HDR for both single target and multiplex genome editing in bacteria and eukaryotic microorganisms (Ronda *et al*. 2015; Ao *et al*. 2018; Wang *et al*. 2018a). In *S. cerevisiae*, multiplex gene deletions can be obtained simultaneously by using oligo-sized dDNAs with short HFs (∼50 bp), either as single-stranded (Generoso *et al*. 2016) or as annealed double-stranded dDNAs (Mans *et al*. 2015; Swiat *et al*. 2017; Ferreira, David and Nielsen 2018) (Table 2). The amount of dDNA has previously been considered a key factor for enhancing CRISPR-Cas-mediated editing (Horwitz *et al*. 2015). Cells are generally transformed with a relatively high concentration of dDNAs, in the order of picomols (Horwitz *et al*. 2015; Mans *et al*. 2015). This concentration is higher than the one usually used for *in vitro* plasmid assembly procedures in *S. cerevisiae* (Kuijpers *et al*. 2013).

### Methods for increasing HDR frequencies versus NHEJ and alternative NHEJ repair

The potential of CRISPR-Cas9/Cas12a for implementing precise HDR-based genome editing is often hindered by (i) the presence of NHEJ or alternative-NHEJ repair systems, (ii) the presence of inefficient HDR systems, or (iii) an unfavorable balance between NHEJ and HDR repair mechanisms.

In bacteria, Cas9 and Cas12a are mainly used as counter-selection tools: the endonucleases create DSBs causing cell death due to the absence or poor efficiency of NHEJ repair systems. Only those cells that successfully obtained and integrated appropriate repair templates into their genome avoid the occurrence of persistent chromosomal breaks (Mougiakos *et al*. 2016). The expression of recombineering systems based on the λ-Red recombinase is extensively used in organisms such as *E. coli*, *Lactobacillus* (Oh and van Pijkeren 2014) or *Pseudomonas putida* (Aparicio, de Lorenzo and Martínez-García 2018) to boost the efficiency of HDR and increase the number of recombinants (Mougiakos *et al*. 2018).

In the case of a multiplex engineering approach in yeasts, the desired genome editing (and the cell viability) relies on the stochastic allocation of dDNAs into the nucleus. In *S. cerevisiae*, the relatively high activity of the HDR machinery facilitates precise editing at multiple loci simultaneously (Mans *et al*. 2015; Swiat *et al*. 2017; Ferreira, David and Nielsen 2018). In contrast, the prevalent NHEJ system in non-conventional yeasts (or other difficult-to-engineer organisms, such as microalgae) might need a more extensive screening due to the high heterogeneity of transformants (Serif *et al*. 2018; Wang *et al*. 2018a). To improve the efficiency of HDR in some yeasts such as *Y. lipolytica*, the *KU70* gene responsible for DSB repair in the NHEJ pathway was disrupted (Jang *et al*. 2018). A similar approach is often applied in non-conventional yeasts, e.g. in *Naumovozyma castellii*, where simultaneous deletion of the orthologues of *KU70* and *KU80* completely abolished NHEJ repair during CRISPR-based editing (Vyas *et al*. 2018). Alternatively, researchers have inhibited certain endogenous DNA repair components to favor HDR in CRISPR experiments (Vyas *et al*. 2018).

The balance between DSB repair pathways is further influenced by the cell cycle phase in which a cell is. By making use of hydroxyurea-mediated cell cycle arrest (S-phase), the frequency of targeted integration is significantly increased in multiple fungi (Tsakraklides *et al*. 2015) and demonstrated specifically for CRISPR-based genome editing of *Y. lipolytica* (Jang *et al*. 2018). Finally, an improvement of HDR efficiency in single target genome editing has recently been achieved by active recruitment of the dDNA to the DSB making use of the ability of some proteins to bind DSBs in *S. cerevisiae* (Roy *et al*. 2018). Similar methods could be considered for obtaining an increased HDR frequency in a multiplexing set-up for a broader variety of microbes.

### Multiplex gene repression and activation

Multiplexing can also be exploited for CRISPRi/CRISPRa approaches (Table 3). Again, both dCas9 and dCas12a have been used for controlling gene expression. In many microorganisms, CRISPRi and CRISPRa have mainly been used to re-direct the carbon flux towards the production of the desired product. This usually requires fine-tuning the expression of multiple genes, which can be achieved by multiplexed gRNA expression for polygenic targeting. Multiplexing using CRISPRi has been explored in several prokaryotic industrial organisms such as *E. coli* (Zhang *et al*. 2017; Gao *et al*. 2018; Tian *et al*. 2019)*, S. coelicolor* (Li *et al*. 2018; Zhao *et al*. 2018), *P. putida* (Tan, Reisch and Prather 2018), *Bacillus subtilis* (Wu *et al*2018b)*, Corynebacterium glutamicum* (Cleto *et al*. 2016) and the cyanobacterium *Synechocystis* (Yao *et al*. 2016; Kaczmarzyk *et al*. 2018). Furthermore, different approaches have been explored in the yeasts *S. cerevisiae* (Jensen *et al*. 2017; Lian *et al*. 2017), *Y. lipolytica* (Schwartz *et al*. 2017; Zhang *et al*. 2018) and *Kluyveromyces marxianus* (Löbs *et al*. 2018). Independent studies on the implementation of Cas12a for CRISPRi purposes reported changes in repression strength after altering the order of the spacers in the CRISPR array (Liao *et al*. 2018). Other studies did not observe the same effect (Wang *et al*. 2017; Zetsche *et al*. 2017; Zhang *et al*. 2017). This suggests that the phenomenon could be gRNA-sequence-dependent and therefore most likely related to transcript secondary structure formation.

**Table 3.** Multiplexed genome regulation events in industrial microorganisms using CRISPRi and CRISPRa.

*Specie* [strain(poidy)]	Cas nuclease tool (CRISPRi/CRISPRa), expression, promoter Plasmid (replication origin)/genome integrated	Strategy for multiplexed gRNA expression/delivery (expression) Plasmid (replication origin)/genome integrated	Goal	Number of targets	Reference
**PROKARYOTES**					
*E. coli*[B0013]	d*Sp*Cas9 (CRISPRi)—constitutive expression, trc promoterPlasmid expression	Several sgRNA expression cassettes (constitutive)Plasmid expression (CloDF13ori)	Increase malate titer	3 targets	(Gao *et al*. 2018)
*E. coli*[MG1655]	dd*As*Cas12a (CRISPRi)—constitutive expression, j23100 promoterPlasmid expression (p15A ori)	Several sgRNA expression cassettes (j23119-SpeI constitutive promoter)Plasmid expression (ColE1 ori)	Proof of principle	3 targets	(Zhang *et al*. 2017)
*E. coli*[MG1655]	d*Sp*Cas9 (CRISPRI/CRISPRa)—constitutive expression, endogenous S. pyogenes promoterPlasmid expression	Several sgRNA expression cassettes (j23119 constitutive promoter) fused to protein binding sequences (scaffold RNAs)Plasmid expression	Proof of principle. Activation of ethanol biosynthesis	2 targets	(Dong *et al*. 2018)
*Bacillus subtilis*[BNY]	d*Sp*Cas9 (CRISPRi)—- inducible expression, *xylA* promoterGenome integrated	Several sgRNA expression cassettes (P_veg_ constitutive promoter)Genome integrated	Increase N-acetylglucosamine titer	3 targets	(Wu *et al*. 2018b)
*Streptomyces coelicolor*	d*Sp*Cas9 (CRISPRi)—constitutive expression, ermE*p promoterGenome integrated	Several sgRNA expression cassettes (j23119p constitutive promoter)Genome integrated	Proof of principle (knock-out 4 pigmented antibiotic synthesis)	3 and 4 targets	(Zhao *et al*. 2018)
*Pseudomonas putida*	d*Spa*Cas9 (CRISPRi)—inducible expression, LacI-P*tac* promoterGenome integrated	Several sgRNA expression cassettes (P*tet* promoter)Plasmid expression (oriV, Rep)	Proof of principle	2 targets	(Tan *et al*. 2018)
*Corynebacterium glutamicum*	d*Sp*Cas9 (CRISPRi)—inducible expression, P*tac* promoterPlasmid expression	Several sgRNA expression cassettes (P*tac* inducible promoter)Plasmid expression	Increase aminoacid production	3 targets	(Cleto *et al*. 2016)
*Bacillus subtilis*[BNY]	d*Sp*Cas9 (CRISPRi)—inducible expression, PxylA promoterGenome integrated	Several sgRNA expression cassettes (P*veg* promoter)Genome integrated	Increase GlcNAc titer	3 targets	(Wu *et al*. 2018)
*Synechocystis*	d*Sp*Cas9 (CRISPRi)—constitutive expression, PpsbA2 promoterGenome integrated	Several sgRNA expression cassettes (P_L31_ constitutive promoter)Genome integrated	Reduction of PHB and glycogen accumulation during nitrogen starvation	4 targets	(Yao *et al*. 2016)
*Synechocystis*	d*Sp*Cas9 (CRISPRi)—inducible expression, P_L22_ promoterGenome integrated	Several sgRNA expression cassettes (P_L22_ constitutive promoter)Genome integrated	Carbon flux re-direction for production of fatty alcohols	6 targets	(Kaczmarzyk *et al*. 2018)
*Streptomyces coelicolor*[M145]	dd*Fn*Cas12a (CRISPRi)—constitutive expression, ermEp* promoterGenome integrated	Native-like CRISPR array (kasOp* constitutive promoter)Genome integrated	Proof of principle (knock-out 3 pigmented antibiotic synthesis)	3 targets	(Li *et al*. 2018)
**EUKARYOTES**					
*Saccharomyces cerevisiae*[CEN.PK2-a and Sigma 10 560–4A]	dCas9-VPR (CRISPRi and CRISPRa)Plasmid expression (centromeric)	TEF1p-tRNA-sgRNA-tRNA (constitutive)Plasmid expression (centromeric)	Increase 2,3-butanediol titer	5 targets (4 interference, 1 activation)	(Deaner, Holzman and Alper 2018)
*Saccharomyces cerevisiae*	d*Sp*Cas9 (CRISPRi/CRISPRa)—inducible expression, pGal10 promoterGenome integrated	Several sgRNA (with RNA scaffolds) expression cassettes (SNR52p constitutive RNA pol III promoter)Plasmid expression (centromeric)	Proof of principle, obtention of different violacein biosynthetic pathway products	3 targets	(Zalatan *et al*. 2015)
*Saccharomyces cerevisiae*	d*Lb*Cas12a-VP (CRISPRa), d*Sp*Cas9-RD1152 (CRISPRi), *Sa*Cas9 (CRISPRd)—constitutive expression, pTDH3 promoterGenome integrated	Several sgRNA and gRNA in synthetic array between Csy4 recognition sites (TEF1p RNA pol II constitutive promoter)Plasmid expression	Increase β-carotene production	3 targets (1 activation, 1 interference and 1 deletion)	(Lian *et al*. 2017)
*Kluyveromyces marxianus*	d*Sp*Cas9 (CRISPRi)—constitutive expression, *TEF1p* promoterPlasmid expression (centromeric)	Several sgRNA expression cassettes (RPR1-tRNA^gly^,RNA pol III constitutive promoter)Plasmid expression (centromeric)	Increase ethyl acetate production	6 targets (4 genes)	(Löbs *et al*. 2018)
*Yarrowia lipolytica*	d*Sp*Cas9-Mxi1 (CRISPRi)—constitutive expression, UAS1B8-TEFPlasmid expression	Several sgRNA expression cassettes (SCR1-tRNA^gly^ RNA pol III constitutive promoter)Plasmid expression	Increase HR by repression of the NHEJ machinery	3 targets (2 genes)	(Schwartz *et al*. 2017)
*Saccharomyces cerevisiae*[BY4741 (n)]	d*Sp*Cas9-VPR (CIRSPRi and CRISPRa)Plasmid expression (centromeric)	Synthetic array of ribozyme-flanked sgRNAs (Gal1p, RNA pol II inducible promoter)Plasmid expression (centromeric)	Proof of principle	2 targets	(Deaner, Mejia and Alper 2017)
*Saccharomyces cerevisiae*[BY4741 (n)]	d*Sp*Cas9-VPR (CIRSPRi and CRISPRa)Plasmid expression (centromeric)	Synthetic array of ribozyme-flanked sgRNAs (TEF1p, RNA pol II constitutive promoter)Plasmid expression (centromeric)	Proof of principle	4 targets	(Deaner, Mejia and Alper 2017)
*Yarrowia lipolytica*[ATCC 201 249]	d*Sp*Cas9 or d*Fn*Cas12a (CRISPRi)—constitutive expressionPlasmid expression (centromeric)	Several sgRNA expression cassettes (SCR1-tRNA^gly^ RNA pol III constitutive promoter)Plasmid expression (centromeric)	Proof of principle, decrease protodeoxy-violaceinic acid	3 targets	(Zhang *et al*. 2018)

Fewer examples can be found of the use of CRISPRa because of the requirement of functional transcription activators. Frequently, CRISPRa applications are used in combination with CRISPRi approaches (Lian *et al*. 2017). Combination of CRISPRi and CRISPRa using RNA scaffolds was accomplished using similar designs in *E. coli* (Dong *et al*. 2018) and *S. cerevisiae* (Zalatan *et al*. 2015; Lian *et al*. 2017). In both cases, protein-binding RNA sequences were fused to the 3′ end of sgRNAs of Cas9. These protein-recruitment RNA sequences have a high affinity for certain proteins, such as the MCP (used both in *E. coli* and *S. cerevisiae*) or the PCP and Com proteins (tested only in *S. cerevisiae*). By fusing the transcriptional activation domain SoxS (in *E. coli*) or VP64 (in *S. cerevisiae*) to these RNA binding proteins, expression of a gene downstream a target promoter can be enhanced, and pathway fluxes sequentially directed (Zalatan *et al*. 2015; Dong *et al*. 2018).

### Challenges and outlook of multiplexing

In recent years, remarkable progress has been achieved in the field of multiplexed genome editing. Besides broadening the spectrum of microorganisms that can be engineered using CRISPR-Cas endonucleases, novel approaches have recently been implemented in terms of gRNA and dDNA delivery for increasing multiplexing efficiency. Below, a summary is provided of the major challenges related to the development of efficient multiplexing CRISPR-Cas systems in microorganisms.

1. *Guide design*: availability of highly efficient guides (sgRNA, crRNA) obtained either through software prediction based on generalized well defined guide-design principles, preferential PAM domains and secondary structure prediction (Chari *et al*. 2015; Graham and Root 2015; Moreno-Mateos *et al*. 2015; Labuhn *et al*. 2018; Liao *et al*. 2018), or through pre-characterization of the functionality of individual guide performance in single target genome editing experiments.
2. *dDNA design and delivery*: dDNA can be part of a vector, provided as a linear fragment, single or double-stranded, a single fragment of multiple fused repair templates or provided as multiple DNA fragments. Proper characterization of such elements (e. g. by determination of optimal size of homology sequences) for the specific host organism to be edited is required. Additionally, dDNA might be stabilized by chemical modifications (Lee *et al*. 2017).
3. *Controllable expression of CRISPR elements*: tight expression control of guides and nucleases, via tuning of expression either by systematic variation of constitutive promoters or by using inducible systems. Optimized promoter and terminator sequences are required to fine-tune the expression of the different CRISPR elements (Feng *et al*. 2018).
4. *Innovative plasmid assembly methods*: smart DNA construction schemes to develop single or multi-plasmid systems containing elements of the CRISPR-Cas system (gRNA, Cas, dDNA, selective marker), including techniques to incorporate repetitive DNA elements such as the repeat sequences of CRISPR arrays (Cress *et al*. 2015; Deaner, Holzman and Alper 2018; Liao *et al*. 2018). Recently, a cloning-free approach was developed in *S. cerevisiae* for the obtainment of up to 6 simultaneous deletions using *Sp*Cas9 with a efficiency of 23.3%. The strategy combined multiple successful strategies presented in this manuscript. Three transcripts (each of them containing two gRNAs flanked by tRNA^gly^ sequences were expressed from a single plasmid encoding for *Sp*Cas9. The assembly of the plasmid via Golden Gate reaction was performed in the yeast (Zhang *et al*. 2019).
5. *Organism-specific CRISPR tools*: smart choice of the CRISPR-Cas expression approach depending on final application of the production strain or the target organism of choice. For instance, CRISPR-Cas tools can be combined with the introduction of dDNA containing selective markers, which makes screening and selection of positive clones more efficient. This approach can be used in proof of principle studies, whereas in other cases, marker-free strains are important. In a similar way, guides and Cas nucleases can be co-expressed from plasmid-borne expression cassettes or expressed sequentially in strains pre-expressing the Cas nuclease from a second plasmid or from a genome integrated copy.
6. *Editing conditions*: optimization of organism-specific CRISPR-Cas delivery systems and recovery protocols. Cell synchronization protocols in combination with CRISPR-Cas systems have been used in human cells to enhance HDR versus NHEJ repair (Lin *et al*. 2014). In some yeasts, the highest rate of HDR over NHEJ is shown during S-phase (Tsakraklides *et al*. 2015). Therefore, this strategy has been already proposed for its use in industrial microorganisms (Juergens *et al*. 2018).
7. *Novel or improved endonucleases*: these endonucleases should have alternative or less-stringent PAM recognition selection. In addition, nucleases are preferred that are smaller, more specific and more active, as reviewed by Kleinstiver *et al*. (2019). Cas9 variants with distinct PAM-recognizing features have been obtained by laboratory evolution (Hu *et al*. 2018), as well as by structure-guided protein engineering of Cas12a (Kleinstiver *et al*. 2019). The discovery and characterization of novel Cas endonucleases (Liu *et al*. 2019)- can also extend the PAM compatibility (Burstein *et al*. 2017; Harrington *et al*. 2018; Strecker *et al*. 2019). At the same time, multiple endonucleases can be used in orthogonal designs in order to edit the genome and regulate gene expression simultaneously (Kweon *et al*. 2017; Lian *et al*. 2017). Alternatively, orthogonal designs can incorporate modified gRNAs, as described for Cas12a by Breinig *et al*. (2019).

In multiplexed genome editing experiments, a negative correlation is experienced between the number of targets and the amount of obtained colonies after transformation in most microorganisms. The introduction of DSBs dramatically reduces the cell survival rate, and this causes limited numbers of simultaneous modifications as a delicate balance between DNA cleavage and repair needs to be established. Alternatively, multiplexed single-base editing does not depend on DSB generation nor dDNA supply and can be used to introduce nucleic acid base changes at a targeted window of DNA (Eid, Alshareef and Mahfouz 2018; Wu *et al*. 2018a). In this approach, deactivated or nickase Cas nuclease variants coupled to base editors (cytidine or adenine deaminases) are directed to a target site by the gRNA (Marx 2018). Nucleotide changes are introduced in a targeted DNA window rather than in a precise DNA position, which makes this technique less accurate for the introduction of single point mutations. Multiplexed single-base editing has been recently implemented in prokaryotic microorganisms (Banno *et al*. 2018; Wang *et al*. 2018b). Useful applications of this technique remain limited to phenotype modifications linked to single nucleotide polymorphisms (SNPs) and to the possibility of introducing stop-codons for gene inactivation in coordination with the presence of a PAM sequence in a determined window (Arazoe, Kondo and Nishida 2018). Although very promising, further development and control of this base editing approach are required in order to broaden the type of modifications and to avoid unwanted mutations (Nishida *et al*. 2016).

Advances in multiplexed genome editing of microorganisms can significantly accelerate future strain construction programs of cell factories with unprecedented efficiencies. Therefore, the CRISPR revolution continues: new tools and workflows are being developed to broaden the range of functionalities of currently used CRISPR-Cas systems as well as knowledge about the mechanism of these systems. As identified in this review, dedicated optimization of each of the elements involved in CRISPR-Cas genome editing is crucial for efficient multiplex genome editing and for stretching the number of simultaneous editing events.
